# Carbamoylated erythropoietin modulates cognitive outcomes of social defeat and differentially regulates gene expression in the dorsal and ventral hippocampus

**DOI:** 10.1038/s41398-018-0168-9

**Published:** 2018-06-08

**Authors:** Monica Sathyanesan, Michael J Watt, Jacob M Haiar, Jamie L Scholl, Shaydel R Davies, Riley T Paulsen, Jayme Wiederin, Pawel Ciborowski, Samuel S Newton

**Affiliations:** 1 Division of basic Biomedical Sciences, Sanford School of Medicine, University of South Dakota, Vermillion, SD 57069 USA; 2 Department of Pharmacology and Experimental Neuroscience, University of Nebraska Medical Center, Omaha, NE 68198 USA; 3 Present Address: Sioux Falls VA Healthcare System, Sioux Falls, SD 57105 USA

## Abstract

Cognitive deficits are widespread in psychiatric disorders and frequently as debilitating as the affective component. Widely prescribed antidepressants for treating depressive disorders have limited efficacy in normalizing cognitive function. Erythropoietin (Epo) has been shown to improve cognitive function in schizophrenia and treatment resistant depressed patients. However, the potent elevation of red blood cell counts by Epo can cause hematological complications in non-anemic patients. We investigated a chemically engineered, posttranslational modification of Epo, carbamoylation, which renders it non-erythropoietic. We conducted mass-spectrometry-based peptide mapping of carbamoylated Epo (Cepo) and tested its ability to improve cognitive function after social defeat stress. Gene expression analysis in discrete brain regions was performed to obtain mechanistic insight of Cepo action. Cepo reversed stress-induced spatial working memory deficits while affecting long-term (24 h) novel object recognition in these rats. Contextual fear conditioning following defeat was enhanced by Cepo, but attenuated in controls. However, Cepo improved fear extinction in all rats compared to vehicle treatment. Cepo induced differential gene expression of BDNF, VGF, Arc, TH. and neuritin in the mPFC and discrete hippocampal subfields, with strongest induction in the dorsal hippocampus. Analysis of gene–brain region–behavior interactions showed that Cepo-induced neurotrophic mechanisms influence cognitive function. Carbamoylated erythropoietin can be developed as a therapeutic neurotrophic agent to treat cognitive dysfunction in neuropsychiatric diseases. Due to its distinct mechanism of action, it is unlikely to cross react with the activity of currently prescribed small molecule drugs and can be used as an add-on biologic drug.

## Introduction

Erythropoietin (Epo), which is widely prescribed to treat anemia, has recently emerged as a potent neurotrophic factor with robust actions on the brain^1^. Several preclinical^2,3^ and clinical studies have demonstrated that systemic administration of Epo is sufficient to produce behavioral effects. Clinical trials have reported striking improvement in cognitive function in schizophrenia patients and treatment resistant depression.

Despite the promising therapeutic effects of Epo in neuropsychiatric disorders, long-term use is likely to cause hematologic complications due to its inherent erythropoietic activity that increases red blood cells. A chemically engineered modification of Epo, carbamoylation, renders Epo non-erythropoietic^4^ but retains the neuroprotective effects^5^. Carbamoylated Epo (Cepo) has been tested in mouse behavioral assays and shows antidepressant-like effects after 28 days of administration and suggested to act by a neurogenic mechanism^6^.

We have previously shown that four days of Epo administration is sufficient to produce behavioral changes in rat and mouse antidepressant-responsive assays^2^. Clinical testing has reported improvement in mood and cognitive function as early as three days after Epo administration^7,8^. As Cepo has been shown to retain Epo’s neurotrophic properties, we hypothesized that it would also improve behavioral performance after a short 4-day administration regimen. We focused our attention on cognition assays as there is a compelling unmet need to effectively treat cognitive deficits in depression^9^. To date, very few preclinical studies have examined the role of Cepo in cognition, and these were performed in healthy rodents without pre-existing cognitive deficits^6,10^. Moreover, the tests employed in these studies were limited to just one aspect of cognitive function, namely long-term declarative memory recall, whereas mood disorders are associated with a much wider range of cognitive deficits.

Therefore, the goal of the current study was to investigate the neurobiology of Cepo’s cognition-enhancing effects by employing the ethologically relevant chronic psychosocial rodent stress model of social defeat, which produces many of the cognitive deficits seen in mood disorders^11–13^. Specific tests examined whether Cepo could alter a range of cognitive outcomes following defeat stress exposure, within paradigms that mimic human cognitive tasks known to be improved by Epo in patients with mood disorders^14–16^. Specifically, performance is increased relative to placebo-treated patients in working memory (digit span and coding test)^17^, declarative memory and object recognition (verbal recall and recognition)^16^, and emotional memory (fearful faces recognition)^18^. Here, we examined effects of Cepo in the rodent equivalents of these tests, namely the delayed spatial T-maze test (working memory)^13^ novel object recognition (declarative memory)^10^ and contextual fear conditioning and extinction (emotional memory)^19^. We produced Cepo in the laboratory and performed molecular characterization by detailed peptide mapping to determine the residues that were modified. The carbamoylated sites were superimposed onto the crystal structure of Epo bound to its receptor in order to obtain structural insight. We analyzed Cepo-induced gene regulation in the medial prefrontal cortex, dorsal, and ventral hippocampus to gain insight into Cepo’s mechanism of action.

## Materials and methods

### Erythropoietin carbamoylation and characterization

Epo was purchased from Prospec Bio (Israel) and carbamoylated in 1 mg aliquots as per^4^, with mild modifications. Briefly, Epo was deprotonated in a high pH (pH = 8.9) borate buffer and then exposed to potassium cyanate for 16 h at 36 °C. Cepo was exhaustively dialyzed for 6 h against PBS. Cepo concentration was determined using the Qubit protein assay (ThermoFisher). Cepo purity was examined using On-chip protein electrophoretic analysis, High Sensitivity Pr0tein 250 chip, run on the Bioanalyzer as per manufacturer’s instructions (Agilent Technologies). Methods for hematocrit measurement and mass-spectrometry analysis are included in the Supplementary material.

#### Peptide mapping and crystal structure superimposition

Cepo was subjected to mass-spectrometry-based peptide mapping to determine the amino acid residues that were modified by carbamoylation. Pure Cepo (see a single, dark band on the electropherogram of Agilent High Sensitivity Protein 250 on-chip gel analysis in Supplementary fig.1) was cleaved by either trypsin or chymotrypsin and subjected to LC-MS/MS analysis on two different MS instruments, a Thermo Orbitrap Elite and Sciex 5600 Triple time of flight MS.

The coordinates for the high resolution X-ray crystal structure of Epo bound to its receptor was obtained from the protein database (PDB ID: 1EER). The three asparagine to lysine substitutions that were introduced for in 1EER^20^ were reversed in PyMOL. Molecular surface of the receptor chains and amino acid residues of the two active sites were generated using the UCSF Chimera package^21^. Side chains of carbamoylated residues were converted to spheres in Chimera.

### Effects of cepo on cognition

#### Animals

Adult male Sprague–Dawley rats (*n* = 40 total, mass mean ± SE = 290 g ± 7.2) were obtained from the University of South Dakota (USD) Animal Resource Center, and pair-housed according to stress treatment group (social defeat or control, *n* = 20 per group) for the duration of the experiments. Larger (>400 g) adult male Sprague–Dawley rats were individually housed and served as aggressive residents for the social defeat procedures, but were not included in any subsequent experimental measures. All rats were maintained at 22 °C on a reverse 12-hr light-dark cycle (lights off at 10:00), with food and water available ad libitum. All behavioral procedures were conducted between 11:00 and 15:00 under red lighting. All procedures were carried out in accordance with the National Institutes of Health Guide for the Care and Use of Laboratory Animals, and received approval from the USD Institutional Animal Care and Use Committee. Every effort was made to minimize the number of animals used and their suffering.

#### Social defeat stress

Subject rats (*n* = 20) were exposed to social defeat each day for five days using a modified resident-intruder paradigm (for details, see^22^ and Supplementary Materials 1). Similar social defeat paradigms have been shown to reduce BDNF expression in the hippocampus, with corresponding deficits in memory performance^11,23^. In addition, we and others have demonstrated that social defeat alters fear conditioning^19,24–26^ and causes deficits in various memory tasks^13,27–30^, outcomes similar to cognitive impairment seen in depressive and schizophrenic patients^17,31^.

#### Cepo administration and behavioral testing

The day following the final social defeat trial, control and defeated rats received single daily injections of either vehicle (PBS, control *n* = 10, defeat *n* = 10) or Cepo (30 µg/kg ip., control *n* = 10, defeat *n* = 10) for 4 consecutive days. All rats then underwent a series of behavioral assays, with novel object recognition training initiated the day after the last injection, followed by a spatial working memory task, and concluding with contextual fear conditioning. The different behavioral tests were separated by a minimum of three days to avoid any carryover effects.

##### Spatial working memory

This task comprised two trials (acquisition and testing) separated by a 30 min inter-trial interval, and used a Plexiglas T maze (start arm 96.8 cm in length, opposing cross arms each 88.4 cm long, all arms 16.3 W × 28 H cm). For the acquisition trial, one of the cross arms was blocked, and the rats were placed into the start arm and allowed to explore the maze for 10 min before being returned to their home cage for the 30 min inter-trial interval. In the test phase, the rats were placed back into the start arm of the T maze and allowed access to all arms for a further 10 min. The entire maze was cleaned with dilute white vinegar and water between the acquisition and test trials to remove any olfactory cues. All trials were videorecorded, with time spent in each arm measured using automated tracking software (Ethovision XT 8, Noldus Information Technology, Leeburg VA USA). A discrimination index ((time in novel arm − time in familiar arm) / (time in novel arm + time in familiar arm)) was also calculated for each subject. Subsequent analyses (see below) revealed no difference in time spent in the start arm at the level of either stress (all controls vs all defeats) or drug treatment (all vehicle vs all Cepo), therefore, calculations of discrimination index in this test did not incorporate time in the start arm.

##### Novel object recognition (NOR)

Long-term non-spatial memory was assessed using the NOR test, with procedures following those described by^10^ as used to demonstrate increased object recognition following short-term Cepo administration. Additional details are provided in the Supplementary Materials 1.

##### Contextual fear conditioning

Detailed description of these procedures can be found in^26^ and Supplementary Materials 1.

#### Gene expression analysis

Two days after the final behavioral experiment, the rats were decapitated and brains rapidly removed, frozen on dry ice, and stored at −80 °C. Frozen brains were sliced coronally at 300 μm using a cryostat (Leica Jung CM 1800; North Central Instruments, Plymouth, MN) and sections thaw-mounted on glass slides and stored at −80 °C. The mPFC (comprising infralimbic, prelimbic, and cingulate cortices), along with subregions of the dorsal and ventral hippocampus (CA1 and dentate gyrus, see Supplementary fig.) were identified using the^32^ rat brain atlas, and microdissected with a 580-μm id cannula using a dissecting microscope and freezing stage (Physiotemp; North Central Instruments). Tissue was collected into RNALater solution for subsequent RNA isolation.

The selection of gene targets was based on previous results of Epo-induced gene regulation, investigated by microarray analysis, and validated by quantitative PCR (QPCR)^2^. Arc was chosen for its well-established role in mediating cognitive function^33^ and TH for the role of dopamine in social defeat^34^. QPCR analysis was performed as previously described^35^.

#### Statistical analysis

##### Gene expression

Relative gene expression using quantitative PCR was calculated using the ΔΔCt method. Data for the individual genes and brain regions were expressed as the inverse of amplification cycle numbers normalized to housekeeping genes and then compared using separate two-way ANOVA (stress × drug) followed by Student–Neman–Keuls tests in SigmaPlot 13.0 (Systat Software Inc., San Jose, California). Gene expression comparisons were considered statistically significant at *p* *<* 0.05.

##### Behavior

For all behavioral data, statistical outliers were identified using separate Grubbs’ tests and removed from analyses. Discrimination indices for the NOR and spatial working memory tests were compared among groups using two-way ANOVA (stress × drug) followed by Student–Newman–Keuls tests when appropriate, and within each group were compared against the theoretical zero value using separate one sample *t*-tests. In addition, time spent in each arm of the T maze used for the working memory task was compared across groups using a three-way mixed design ANOVA (stress × drug × repeated measure of arm), with significant interactions followed by two-way repeated measures ANOVA and SNK tests. For contextual fear learning, time spent freezing was compared across sessions (Recall, Extinction 1, Extinction 2) using three-way mixed design ANOVA (stress × drug × repeated factor of session), with effects of session further investigated using one-way repeated measures ANOVA within each treatment group followed by Holm–Sidak tests, and significant stress × drug × session interactions followed by separate two-way ANOVA across groups within each session. Similarly, expression of freezing over time within the Recall and Extinction 1 sessions was also analyzed separately using three-way mixed design ANOVA (stress × drug × repeated measure of time), followed by one-way repeated measures ANOVA within each group and two-way ANOVA at each time point where appropriate. All data for three-way mixed model ANOVA were adjusted for non-sphericity using a Greenhouse-Geisser correction where necessary, as indicated when degrees of freedom are reported in decimal format (analyses meeting sphericity have degrees of freedom given in whole numbers), and were conducted using SPSS Statistics 24 (IBM Corp., Armonk, New York). Further ANOVA and post-hoc tests for multiple comparisons were completed using SigmaPlot v13.0. The alpha level was set at 0.05 throughout.

##### Modeling gene × behavior relationships

Potential relationships between changes in gene expression within each brain region and behavior were examined using separate multiple linear regressions, with specific genes then removed systematically to identify the gene combination providing the best linear fit (see Supplementary Table 1). In addition, we performed multiple regressions to examine how gene expression across numerous brain regions could exert a combined effect on each behavior of interest (see Supplementary Material 1). All regressions were performed using SigmaPlot 13.0 at an alpha level of 0.05.

## Results

### Cepo characterization

**Fig. 1:**
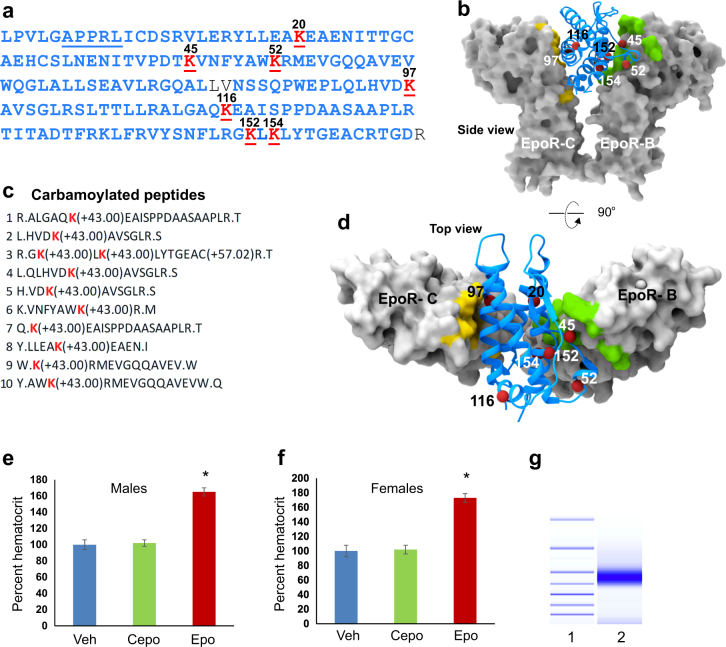
Peptide mapping of erythropoietin carbamoylation. **a** The amino acid sequence of carbamoylated erythropoietin obtained by mass-spectrometry mapping of trypsin and chymotrypsin peptides is shown bolded in blue. Residues that were not detected are shown in regular black font. The start of the mature sequence is underlined. Carbamoylated lysine residues are shown in red with position number above. **b** The position of carbamoylated residues are shown superimposed upon the crystal structure of Epo bound to EPOR (PDB ID: 1EER). EPOR dimers are show in the molecular surface configuration and Epo secondary structure in blue. Carbamoylated residues are indicated by red spheres. The molecular surface of residues that comprise the high affinity, active site 1 is colored green and the low affinity, active site 2 in yellow. **c** A short list of carbamoylated peptides with modified residues in red, followed by the detected change in mass. **d** The side view in (**b**) is rotated 90° to provide an extracellular top view. Cepo is non-erythropoietic in males and females. Epo and Cepo were tested in parallel for erythropoietic activity at a dose of 30 μg/kg/day for 10 days in (**e**) male and (**f**) female BALB/c mice. Bar graph represents the mean of *n* = 6. Error bars are SEM, *t*-test (*p < 0.05). **g** Cepo purity examined using High Sensitivity Protein 250 chip (Agilent), Lane 1 = markers, lane 2 = Cepo

Employing two different proteolytic enzymes, trypsin and chymotrypsin, we obtained amino acid sequence information for 180 Cepo peptides in our mass-spectrometry analysis. This resulted in high overall coverage of the entire molecule, only three amino acid residues (in black font) were missed (Fig. 1a). Carbamoylation was determined by the presence of the carbamoyl moiety, consisting of carbon, hydrogen, nitrogen, and oxygen (CHNO) atoms, yielding a mass of 43 Da (Fig. 1c). Residues that were consistently carbamoylated were all lysines, shown in red and numbered. A short list of carbamoylated peptides is shown in Fig. 1c. The full list is in Supplementary Materials 2. The only lysine that was not carbamoylated is in position 140. Occasionally (7 of 180 peptides), we noted carbamoylation of arginine residues at positons 53, 111, and 131. The N-terminus was also carbamoylated. The four cysteine residues present in Epo underwent carbamidomethylation, showing a 57 Da increase in mass, while methionine residues were oxidized, evidenced by the 16 Da increase. The physical location of carbamoylated lysine residues in Cepo are shown as red spheres (Fig. 1b, d) in the high resolution crystal structure of Epo bound to EPOR (PDB ID: 1EER). The residues that comprise the high affinity and low affinity binding sites of Epo receptor are shown in green and yellow, respectively, in the molecular surface representation (Fig. 1b, d). The cell surface, top view (Fig. 1d) shows that five of the seven carbamoylated lysine residues of Cepo face the high affinity site on chain B of EPOR. Only two modified residues are on the helix that faces the low affinity site on chain C (Fig. 1d).

We tested Cepo for erythropoietic activity by administering it for 10 days to male and female mice (Fig. 1e, f). While Epo robustly elevated hematocrit in males (Fig. 1e) and females (Fig. 1f), Cepo had no effect on hematocrit, and the levels were comparable to vehicle-treated mice (Fig. 1e, f). We also tested the purity of Cepo that we generated by high sensitivity electrophoretic analysis on a biofluidics instrument. A prominent single band, indicative of high purity, was detected (Fig. 1g).

### Effects of cepo on cognition

#### Spatial working memory

Regardless of drug administration, all control and defeated rats appeared able to distinguish the novel arm from the familiar arm (F2,69 = 3.19, *P* = 0.044, $\eta _p^2$ = 0.087, SNK *P* < 0.001, Hedge’s *g* = 1.10–1.83), but time spent in the start and novel arms did not differ as a function of stress, although there was a trend for all defeated rats to spend less time in the familiar arm (SNK *P* = 0.07, Hedge’s *g* = 0.46).

**Fig. 2:**
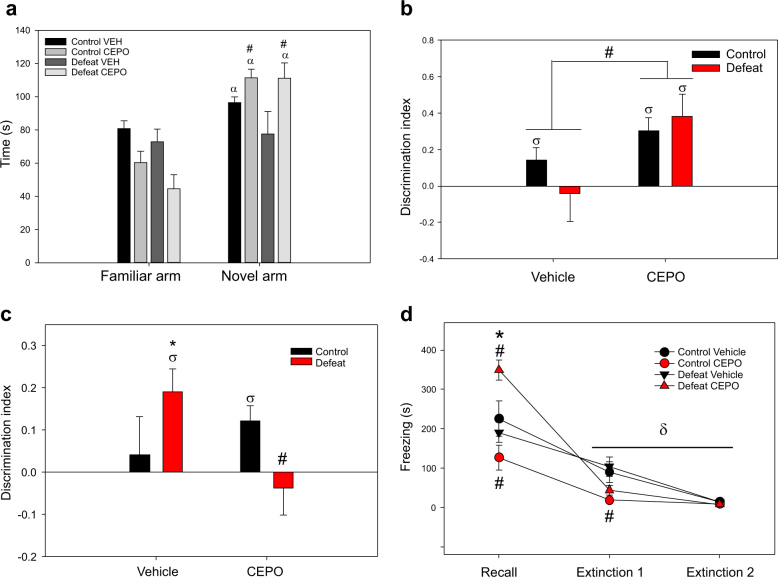
**a** Prior stress and repeated Cepo have differential effects on time spent in each arm during the T-maze spatial working memory task. Only defeated rats that received vehicle failed to show a difference in exploration of novel vs familiar arms, while Cepo treatment increased time spent in the novel arm regardless of prior stress experience. α Novel > familiar within each group, # effect of drug. **b** Arm discrimination during spatial working memory task. Discrimination indices for the T-maze spatial working memory task are significantly greater than the theoretical zero random choice level for all groups, except for vehicle-treated defeated rats. Administration of Cepo also increased discrimination indices relative to vehicle treatment. σ Higher than zero random choice level within each group, # effect of drug across groups. *N* = 8–10/group. **c** Ability to discriminate objects during the test phase of the novel object recognition task (24 h after acquisition phase) is greater in vehicle-treated defeated rats compared to their control counterparts, which is also reflected by a discrimination index higher than the theoretical zero choice level in this group. However, object discrimination rats exposed to social defeat is impaired by Cepo treatment. In contrast, Cepo shows a trend for improving performance in control rats, elevating the discrimination index significantly above chance levels. σ Higher than zero random choice level within each group, * effect of stress across groups, # effect of drug across groups. *N* = 8–10/group. **d** All rats exhibited contextual fear learning and extinction, as indicated by high levels of conditioned freezing during the Recall session (24 h after acquisition) that declined with subsequent exposure to the fearful context. However, treatment with Cepo produced divergent effects depending on both prior stress exposure and testing day. During Recall, conditioned freezing was augmented by Cepo in defeated rats and blunted in controls, but during the first Extinction session, all Cepo-treated rats showed lower freezing than those receiving vehicle. δ Different from Recall within each group, * effect of stress across groups, # effect of drug across groups. *N* = 8–10/group

Drug treatment had effects on time spent in each arm separate from those caused by prior stress (F2,60 = 10.26, *P* < 0.001, $\eta _p^2$ = 0.23). All Cepo-treated subjects spent more time in the novel than the familiar arm (Fig. 2a, SNK *P* < 0.001, Hedge’s *g* = 2.53), whereas this effect appeared to be absent in vehicle-treated rats (Fig. 2a). In addition, investigation of the novel arm was significantly higher in Cepo-treated rats than in vehicle groups (Fig. 2a, SNK *P* = 0.004, Hedge’s *g* = 1.0). There was no difference between time spent in the start arm between vehicle- and Cepo-treated rats (not shown). The effect of Cepo on increasing time in the novel arm was reflected in differences in discrimination index (time (novel – familiar) / time (novel + familiar)), with two-way ANOVA (stress × drug) revealing a main effect of drug (F1,36 = 7.8, *P* = 0.008, $\eta _p^2$ = 0.043), such that all Cepo-treated rats exhibited higher discrimination indices than vehicle-treated subjects (Fig. 2b).

While a lack of a stress × drug × arm interaction in the initial three-way mixed design ANOVA precluded direct comparisons among the four treatment groups, separate one-way repeated measures ANOVA pointed to differences in ability to distinguish the novel from familiar arm within each group. Vehicle-treated control rats spent more time in the novel compared to familiar arm (Fig. 2a, F2,16 = 16.3, *P* < 0.001, $\eta _p^2$ = 0.72, SNK *P* = 0.041, Hedge’s *g* = 1.23), as did Cepo-treated control (F2,18 = 33.45, *P* < 0.001, $\eta _p^2$ = 0.79, SNK < 0.001, Hedge’s *g* = 2.7) and Cepo-treated defeated rats (F2,15 = 29.18, *P* < 0.001, $\eta _p^2$ = 0.78, SNK *P* < 0.001, Hedge’s *g* = 2.4) (Fig. 2a). In contrast, while there was an effect of arm type in defeated rats that received vehicle (F2,16 = 17.43, *P* < 0.001, $\eta _p^2$ = 0.67), this was solely a function of greater time spent in the start arm (SNK *P* < 0.001, Hedge’s *g* > 2.05), with this group failing to show a difference in exploration of familiar vs novel arms (Fig. 2a). This was supported by one sample *t*-tests comparing discrimination indices within each group, with significantly greater values than the theoretical zero (i.e., no difference in time spent exploring novel vs familiar objects) being seen in all groups except for vehicle-treated defeated rats (Fig. 2b, t9 = −0.29. *P* = 0.78, Hedge’s *g* = −0.09), suggesting experience of social defeat had a negative effect on spatial working memory that was ameliorated by Cepo.

#### Long-term memory

Differences among treatment groups were only apparent during the test phase of the NOR task, with discrimination indices varying as a function of both prior stress and drug (Fig. 2c, F1,31 = 5.59, *P* = 0.025, $\eta _p^2$ = 0.34). Contrary to predictions, vehicle-treated defeated rats had better NOR than their control counterparts (Fig. 2c, SNK *P* = 0.021, Hedge’s *g* = 1.0). In addition, Cepo administration had divergent effects depending on prior stress exposure. Specifically, defeat + Cepo rats showed impaired performance compared to defeat + vehicle rats (Fig. 2c, SNK *P* = 0.012, Hedge’s *g* = −1.2). In contrast, there was a trend for Cepo to improve NOR in controls compared to vehicle treatment (Fig. 2c, SNK *P* = 0.07, Hedge’s *g* = 0.34). Separate one sample *t*-tests for each group against the theoretical zero value (Fig. 2c) showed that NOR was significantly above chance level in defeat + vehicle (t8 = 5.45, *P* < 0.001, Hedge’s *g* = 1.15) and control + Cepo (t8 = 6.25, *P* < 0.001, Hedge’s *g* = 2.36) rats, but not in either control + vehicle (t7 = −0.21, *P* = 0.84, Hedge’s *g* = −0.074) or defeat + Cepo (t8 = −0.54, *P* = 0.6, Hedge’s *g* = −0.19).

##### Contextual fear conditioning

Prior stress experience, drug treatment and session (Fig. 2d) all interacted to influence conditioned fear (F2,62 = 6.81, *P* = 0.002, $\eta _p^2$ = 0.18). Separate one-way repeated measures ANOVA within each group showed that all rats exhibited the greatest amount of freezing during the Recall session relative to subsequent Extinction sessions (Fig. 2d, *P* < 0.001, $\eta _p^2$ = 0.64–0.96).

##### Comparisons of freezing across sessions

Cepo administration had differential effects on freezing according to prior stress exposure, but only during the Recall session (Fig. 2d, F1,33 = 14.8, *P* < 0.001, $\eta _p^2$ = 0.31). Specifically, freezing during Recall was decreased in Cepo-treated control rats relative to both vehicle-treated controls (SNK *P* = 0.042, Hedge’s *g* = 0.75) and defeat + Cepo rats (SNK *P* < 0.001, Hedge’s *g* = 2.45). Cepo administration had the opposite effect in defeated rats, with this group showing higher amounts of freezing compared both to defeat + vehicle (SNK *P* = 0.002, Hedge’s *g* = −2.45) and to Cepo-treated controls (SNK *P* < 0.001, Hedge’s *g* = 1.98). Freezing during Recall was equivalent in vehicle-treated control and defeated subjects (Fig. 2d).

On the first session of Extinction (Fig. 2d), there was only a main effect of drug (F1,32 = 10.37, *P* = 0.003, $\eta _p^2$ = 0.25), but no stress effect or stress x drug interaction, with all Cepo-treated rats displaying lower amounts of freezing compared to vehicle. This drug effect did not persist to the following day, with all groups showing equivalent amounts of freezing during the second Extinction session (Fig. 2d). Extinction learning 24 h later appears to be accelerated in all Cepo-treated animals compared to those receiving vehicle.

##### Comparisons of freezing within sessions

**Fig. 3:**
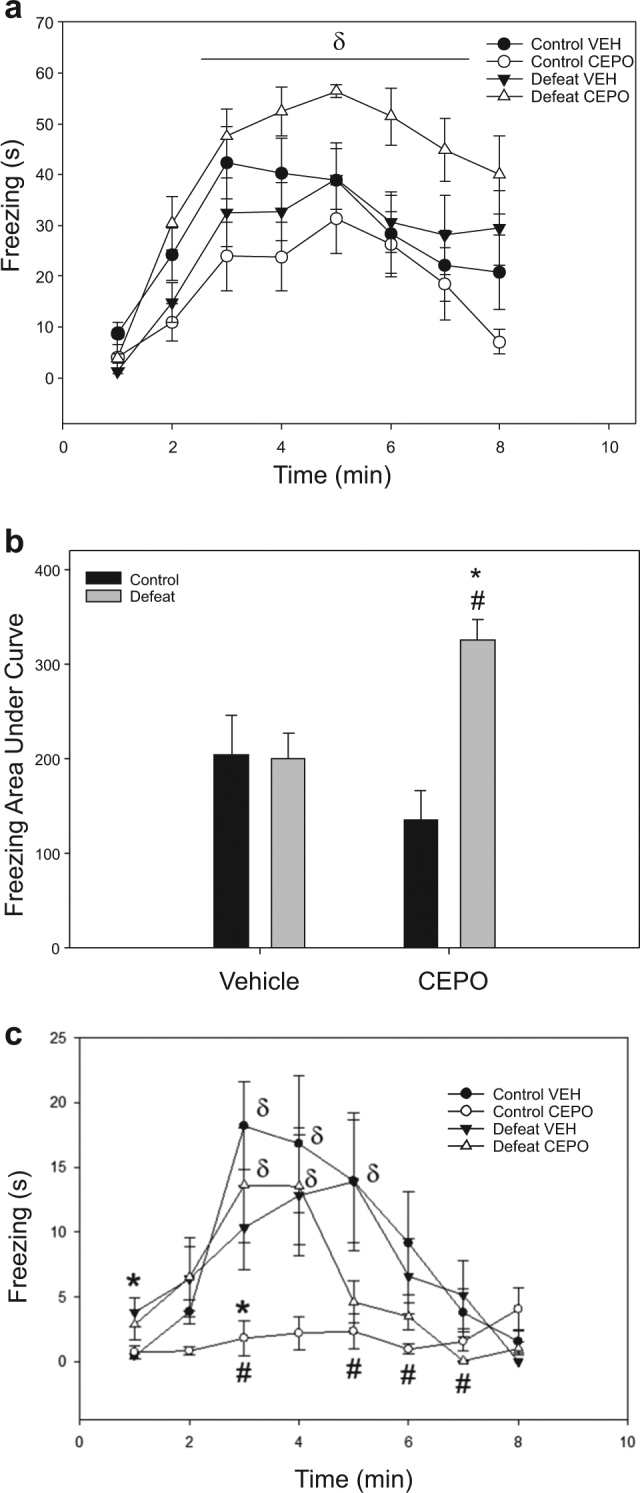
Conditioned fear expression within sessions. All groups showed an increase in freezing across minutes within the Recall session (**a**). The total amount of freezing (calculated as Area Under Curve) was greatest in defeated rats that also received Cepo (**b**). However, defeated rats given Cepo appear to extinguish conditioned freezing during the first Extinction session sooner than their vehicle-treated counterparts (**c**). δ Different from first minute, *effect of stress, #effect of drug

Effects of Cepo according to prior stress were also evident when examining freezing expression at each minute within the Recall session (Fig. 3a). A stress × time interaction was evident (F3.43, 113.14 = 2.81, *P* = 0.036, $\eta _p^2$ = 0.079), with all rats showing increased freezing as the session progressed (Fig. 3a, Holm–Sidak *P* < 0.001). There was also a separate stress × drug interaction (F1,33 = 14.81, *P* = 0.001, $\eta _p^2$ = 0.31), with comparison of area under the curve by two-way ANOVA (Fig. 3b, F1,33 = 9.35, *P* = 0.004, $\eta _p^2$ = 0.21) showing that defeat + Cepo rats displayed higher amounts of freezing as time progressed during Recall than either defeat + vehicle (SNK *P* = 0.01, Hedge’s *g* = 1.52) or control + Cepo rats (SNK *P* < 0.001, Hedge’s *g* = 2.13). Freezing over time within the Recall session did not differ between vehicle-treated control and defeated rats, nor between control + vehicle and control + Cepo rats (Fig. 2b). Combined, this suggests Cepo administration maintains within-session conditioned freezing in previously defeated rats, but does not accelerate within-session extinguishing of the conditioned response in controls.

In contrast to the Recall session, freezing expression across time within the first Extinction session (Fig. 3c) was influenced by a stress × drug × time interaction (F3.51,84.15 = 2.71, *P* = 0.034, $\eta _p^2$ = 0.11). Vehicle-treated controls exhibited an increase and decline in conditioned freezing across time (Fig. 3c, F9,52 = 5.85, *P* < 0.001, $\eta _p^2$ = 0.5) during Extinction 1, with responses being higher in minutes 3 to 5 compared to the first minute of context exposure (Fig. 3c, Holm–Sidak *P* < 0.01). A similar pattern was seen in defeat + Cepo rats (Fig. 3c, F8,49 = 5.43, *P* < 0.001, $\eta _p^2$ = 0.46), with increased freezing restricted to minutes 3 through 4 (Holm–Sidak *P* < 0.034). In contrast, despite a significant main effect of time in defeat + vehicle rats (F10,54 = 3.08, *P* = 0.008, $\eta _p^2$ = 0.36), this group showed no significant change in freezing when compared to the first minute (Fig. 3c), while control + Cepo rats showed no change in freezing expression across the entire session (Fig. 3c, F9,53 = 1.27, *P* = 0.28, $\eta _p^2$ = 0.17).

Comparisons of freezing at each minute within Extinction session 1 showed that effects of social defeat dominated in the first minute (F1,33 = 9.84, *P* = 0.004, $\eta _p^2$ = 0.24), with defeated rats displaying higher amounts of freezing than controls regardless of drug treatment (Fig. 3c). A small trend for a similar effect of defeat was seen in the second minute (F1,31 = 3.86, *P* = 0.058, $\eta _p^2$ = 0.11), whereas drug administration had the major influence on freezing from minutes 5 through 7 (SNK *P* < 0.03), such that Cepo decreased freezing relative to all vehicle-treated rats at these later time points (Fig. 3c). Stress × drug interactions were only apparent in the third minute (F1,33 = 8.06, *P* = 0.008, $\eta _p^2$ = 0.2), with control + Cepo rats showing decreased freezing at this time point (Fig. 3c, SNK *P* = 0.003 vs vehicle, Hedge’s *g* = 1.85; SNK = P 0.024 vs defeat + Cepo, Hedge’s *g* = 1.04). Equivalent levels of freezing during the third minute were seen among control + vehicle, defeat + vehicle, and defeat + Cepo rats (Fig. 3c). These results suggest that Cepo not only has a general dampening effect on conditioned fear within a second exposure to the fearful context, but also possibly indicates Cepo enhances rapid acquisition of extinction learning across the span of minutes in pre-stressed animals, despite their showing greater conditioned fear 24 h previously.

#### Stress- and Cepo-induced gene regulation

##### Dentate gyrus (DG)

**Fig. 4:**
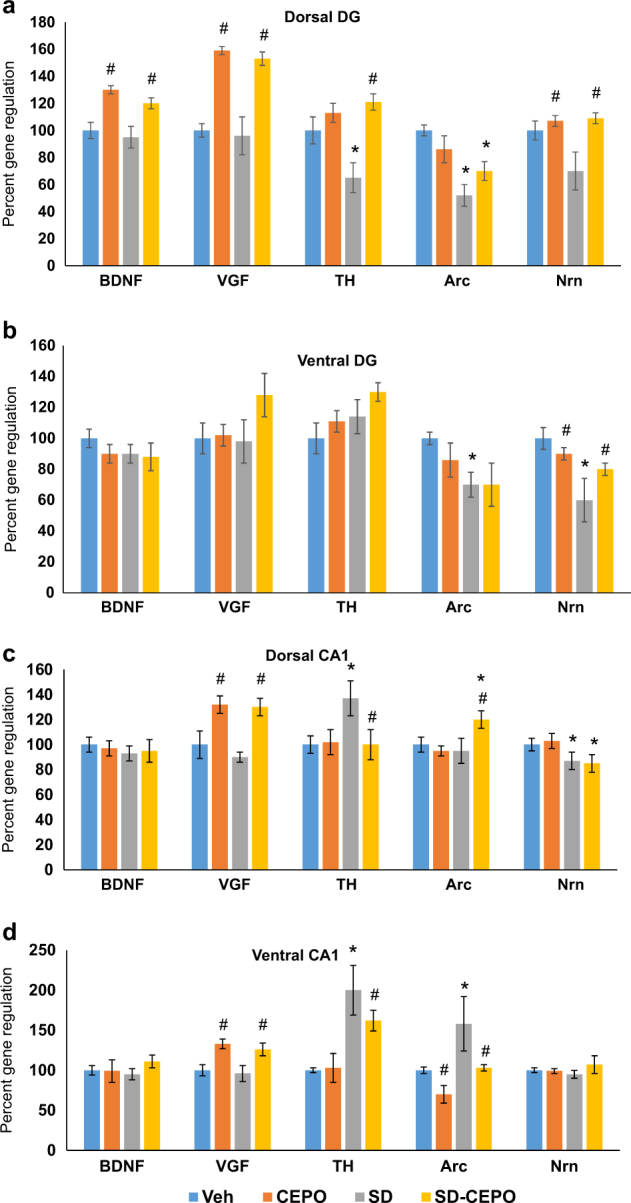
Cepo-induced hippocampal gene regulation. **a** Gene expression changes in the rat dorsal dentate gyrus measured by quantitative PCR. **b** Gene expression changes in the ventral dentate gyrus. **c** Gene expression changes in the rat dorsal CA1 measured by quantitative PCR. **d** Gene expression changes in the ventral CA1. Bars represent the mean of *n* *=* 8. Error bars are ± SEM; *p* *<* 0.05, two-way ANOVA followed by Student–Newman–Keuls tests, *effect of stress #effect of drug. BDNF brain-derived neurotrophic factor, VGF non-acronymic, TH tyrosine hydroxylase, Arc activity regulated cytoskeleton-associated protein, Nrn neuritin. Veh vehicle group, Cepo carbamoylated erythropoietin group, SD social defeat group, SD-Cepo social defeat group administered Cepo

In both control and defeated rats, Cepo upregulated BDNF up by 30% (two-way ANOVA F1,28 = 16.2, *P* < 0.001, $\eta _p^2$ = 0.37), VGF up by 60% (F1,28 = 88.15, *P* < 0.001, $\eta _p^2$ = 0.76), and neuritin (F1,28 = 16.6, *P* < 0.001, $\eta _p^2$ = 0.37) compared to vehicle treatment (Fig. 4a). In contrast, Arc expression in the dorsal DG was downregulated by exposure to social defeat (F1,28 = 18.06, *P* < 0.001, $\eta _p^2$ = 0.39), but was not affected by Cepo (Fig. 4a). Interactions between prior stress and drug influenced TH expression in the dorsal DG (F1,28 = 14.13, *P* < 0.001, $\eta _p^2$ = 0.2), with the downregulation in TH expression caused by social defeat (SNK *P* < 0.001 vs control vehicle, Hedge’s *g* = 1.97) being restored to control levels by Cepo (Fig. 4a).

In the ventral DG, changes to gene expression were restricted to Arc and neuritin (Fig. 4b). While there was a weak interaction between stress and drug for Arc (F1,28 = 4.48, *P* = 0.043, $\eta _p^2$ = 0.14), this was limited to vehicle-treated groups, with defeated rats exhibiting a downregulation in Arc relative to controls (SNK *P* = 0.001, Hedge’s *g* = 1.86) and Cepo having no effect in either group (Fig. 4b). Neuritin expression in the ventral DG was strongly affected by a stress × Cepo interaction (F1,28 = 8.71, *P* = 0.006, $\eta _p^2$ = 0.24). Within vehicle-treated subjects, defeat exposure decreased ventral DG neuritin expression (SNK *P* < 0.001, Hedge’s *g* = 2.32), while within control rats Cepo caused a small but significant reduction compared to vehicle (SNK *P* = 0.048, Hedge’s *g* = 1.17). Similar to TH in the dorsal DG, the defeat-induced downregulation in ventral DG neuritin (SNK *P* = 0.04 vs control, Hedge’s *g* = 1.06) was reversed by Cepo (Fig. 4b). There were no changes to BDNF, VGF, or TH in the ventral DG.

##### Hippocampal CA1

Expression of all genes was altered in the dorsal CA1 region of the hippocampus, with the exception of BDNF (Fig. 4c). Levels of VGF were upregulated 32% in all Cepo-treated rats (F1,28 = 22.95, *P* < 0.001, $\eta _p^2$ = 0.45), while neuritin was downregulated in all defeated rats (F1,28 = 10.67, *P* = 0.003, $\eta _p^2$ = 0.28). Defeat exposure and Cepo interacted to change expression of TH (F1,28 = 4.19, *P* = 0.048, $\eta _p^2$ = 0.13) and Arc (F1,28 = 4.72, *P* = 0.038, $\eta _p^2$ = 0.14) in the dorsal CA1. Social defeat markedly increased levels of dorsal CA1 TH (SNK *P* = 0.014 vs control, Hedge’s *g* = 1.34), which were normalized by Cepo (Fig. 4c). In contrast, Cepo caused an elevation in expression of Arc in the dorsal CA1 of defeated rats (Fig. 4c, SNK *P* = 0.024 vs vehicle, Hedge’s *g* = −1.0).

In the ventral CA1, changes in VGF and TH expression reminiscent of those seen in the dorsal CA1 were evident (Fig. 4d), with VGF being upregulated by Cepo regardless of prior stress (F1,28 = 30.76, *P* < 0.001, $\eta _p^2$ = 0.52). Stress and Cepo interacted to affect TH expression (F1,28 = 16.24, *P* < 0.001, $\eta _p^2$ = 0.37), with increases in TH caused by social defeat (SNK *P* < 0.001 vs control, Hedge’s *g* = 3.16) showing a trend for being lowered by Cepo (SNK *P* = 0.07 vs vehicle, Hedge’s *g* = 1.3). Expression of Arc in the ventral CA1 was independently affected by social defeat (F1,28 = 9.22, *P* = 0.005, $\eta _p^2$ = 0.25) and Cepo (F1,28 = 7.67, *P* = 0.01, $\eta _p^2$ = 0.22), such that Arc was upregulated in defeated rats (SNK *P* = 0.018 vs control, Hedge’s *g* = 0.95) but lowered by Cepo (SNK *P* = 0.027 vs vehicle, Hedge’s *g* = 0.89). No change to either BDNF or neuritin was observed in the ventral CA1 (Fig. 4d).

##### Medial prefrontal cortex (mPFC)

**Fig. 5:**
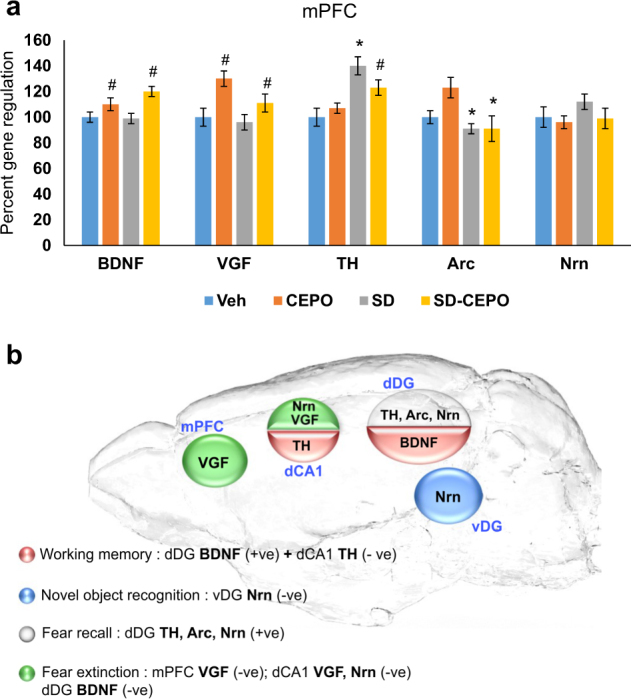
Cepo-induced mPCF gene regulation and gene-brain region-behavior interaction. **a** Gene expression changes in the rat medial prefrontal cortex (mPFC) measured by quantitative PCR. Bars represent the mean of *n* *=* 8. Error bars are ± SEM; *p* *<* 0.05, two-way ANOVA followed by Student–Newman–Keuls tests, *effect of stress #effect of drug. BDNF brain-derived neurotrophic factor, VGF non-acronymic, TH tyrosine hydroxylase, Arc activity regulated cytoskeleton-associated protein, Nrn neuritin. Veh vehicle group, Cepo carbamoylated erythropoietin group, SD social defeat group, SD-Cepo social defeat group administered Cepo. **b** Gene–brain region–behavior interaction. Interactions between regulated genes from gene expression analysis in particular brain regions and behavioral assays are schematically depicted. The contributions of genes and brain region to a particular behavior are classified as positive or negative

The mPFC exhibited changes in expression of all genes following either social defeat or Cepo treatment, with the exception of neuritin (Fig. 5a). Like in the dorsal DG, levels of both BDNF (F1,28 = 9.76, *P* = 0.004, $\eta _p^2$ = 0.26) and VGF (F1,28 = 10.47, *P* = 0.003, $\eta _p^2$ = 0.27) were increased by Cepo, with social defeat having no effect on either gene. In contrast, Arc expression in the mPFC was downregulated in all socially defeated rats (F1,28 = 5.08, *P* = 0.032, $\eta _p^2$ = 0.15) regardless of Cepo treatment. Again similar to hippocampal regions, social defeat and Cepo interacted to affect TH in the mPFC (F1,28 = 6.14, *P* = 0.02, $\eta _p^2$ = 0.18), being upregulated by social defeat (SNK *P* < 0.001 vs control, Hedge’s *g* = −2.05) but normalized by Cepo treatment (SNK *P* = 0.016 vs vehicle, Hedge’s *g* = −1.2).

#### Within brain region gene × behavior relationships

Results of multiple regressions providing models of best fit for variance in each behavior against gene expression within specific brain regions are shown in Supplementary Table 3. In some cases, combinations of two or more genes provided the strongest models, even when only one of the genes was a significant predictor, i.e., relationships between the single gene and behavior were strengthened when other non-significant genes were included, suggesting interaction among genes in mediating behavior.

#### Across brain region gene × behavior relationships

Multiple regressions within regions showed that only neuritin in the ventral DG was associated with novel object recognition (Fig. 5b), while conditioned fear in the Recall session appeared be reflected by gene expression (TH, Arc, and neuritin) only in the dorsal DG (Fig. 5b; Supplementary Table 3). Therefore, multiple regressions across regions were not carried out for these two behaviors. For working memory, the combination of dorsal DG BDNF and dorsal CA1 TH provided the strongest model to account for variance in behavior (Fig. 5b, R2 = 0.43, F2,28 = 10.36, *P* < 0.001), with dorsal DG BDNF (*P* = 0.002) and dorsal CA1 TH (*P* = 0.013) being positively and negatively correlated, respectively, with performance in this task. Variance in fear extinction was best explained by a combination of dorsal CA1 VGF + Nrn and mPFC VGF (Fig. 5b, R2 = 0.48, F 3,26 = 7.98, *P* < 0.001), with all candidates being negatively correlated with the amount of freezing (dorsal CA1 VGF *P* = 0.017, dorsal CA1 Nrn *P* = 0.023, mPFC VGF *P* = 0.01).

## Discussion

We conducted a mass-spectrometry-based mapping of Cepo and investigated its ability to modulate behavioral outcomes of the social defeat paradigm. We also examined Cepo-induced gene regulation in multiple brain regions. As expected, carbamoylation was consistently observed in lysine residues. Cyanate is known to convert lysine to epsilon-N-carbamoyl lysine^36^. It also occurred on amino-terminus residues, and infrequently in certain arginine residues. Most of the modified lysine residues face the B chain of the Epo receptor, with K45 and K20 also being in close proximity to the high affinity active site residues. K97 is the only carbamoylated lysine that is close to the low affinity site. However, it is in closer proximity to a receptor amino acid (2.7 A° from EPOR Glu34) than all the other carbamoylated lysines^20^. Given the crucial location of the carbamoylated residues, it is tempting to speculate that Cepo influences the angular orientation of the receptor dimers^20,37^, or potentially binds to an alternate receptor, leading to a heteromer configuration consisting of EPOR and the betacommon receptor monomers^38^.

Although the precise mechanism involved in Cepo’s lack of erythropoietic activity is not understood, it is useful to note that the neurotrophic genes, BDNF, VGF, and neuritin, which were induced by Epo^2^ are also upregulated by Cepo. This suggests that Cepo retains the neurotrophic properties of Epo without influencing hematocrit. In addition to being induced by Epo, BDNF, VGF, and neuritin are also regulated by electroconvulsive seizure (ECS)^39^ and exercise^40^. These neurotrophic molecules are likely to be involved in the antidepressant effects of ECS and exercise as they have been demonstrated to independently produce antidepressant-like effects after infusion into the brain or viral-mediated overexpression^40–42^. Interestingly, they are also linked with improving cognitive function^43–45^. Promising results from clinical trials indicate that Epo has both antidepressant and cognitive enhancing effects^15,16^. Magnetic resonance imaging studies have reported that Epo is capable of reversing reductions in hippocampal volume^46^, suggesting that it could be an important site of Epo’s actions in the brain.

The Cepo-induced upregulation of BDNF and VGF was higher in the dorsal hippocampal cell layers in comparison to the ventral hippocampal layers. Based on the expression of these genes and the understanding that the hippocampus is functionally divided into a dorsal region that is primarily engaged in cognitive function and a ventral region that regulates emotion^47^, our results provide support for the clinical observations suggesting that Epo has both cognitive enhancing and antidepressant effects^16^. The reduction in Arc levels in the ventral CA1 of Cepo-treated animals mirrors the Arc reduction reported in mice resilient to social defeat, pointing to a potential antidepressant mechanism^48^. Furthermore, defeated rats in our study exhibited a sharp increase in ventral CA1 Arc, which was reversed by Cepo. However, Arc is a molecule that is centrally involved in cognitive mechanisms^33^. Recent work has demonstrated that the induction of Arc in the mPFC and the dorsal hippocampus is essential for memory enhancement^49^. In our study Arc was downregulated in both the dorsal dentate gyrus and mPFC by social defeat, similar to the decrease in Arc seen in the mPFC of social defeat stress susceptible mice^48^.

Multiple regression analyses provided further support for a functional interaction between changes in gene expression and behavior. Interestingly, combinations of two or more genes provided the strongest models in some situations, even when only one of the genes was a significant predictor, suggesting some level of interaction among genes in mediating behavior. For instance, it was found that a combination of increases in both BDNF and TH in the dorsal DG explained better working memory performance, even though only BDNF contributed significantly to the model. Variance in behavior was in some cases predicted by two opposing models incorporating different genes, such as BDNF, TH and Arc in the dorsal DG being positively correlated with the degree of freezing during fear extinction, while a negative association was evident when a combination of VGF, Arc and neuritin from the same region was employed.

Some of the strongest associations between changes to gene expression and behavior were seen in working memory. Defeat exposure produced working memory deficits, which was correlated with low BDNF and TH in the dorsal DG and reduced VGF in the ventral CA1, but increased TH in the dorsal CA1. The result for the dorsal CA1 TH conflicts with recent studies showing that optogenetic stimulation of TH(+) terminals projecting from the locus coeruleus to the CA1 is associated with improved memory consolidation^50^, while lesioning TH(+) terminals in the CA1 produces deficits specific to spatial memory tasks^51^. It is possible that the increase in TH mRNA expression in the dorsal CA1 of defeated rats represents overcompensation for decreased TH that may have been evident immediately after the stress experience. Relatively little is known about the role of TH in the DG with regards to memory performance. There however appears to be a positive relationship between neurotrophic factors in the dentate gyrus and TH, with application of glial-derived neurotrophic factor increasing neuronal TH^52^, and BDNF in the dentate gyrus with a well-characterized role in long-term potentiation that is thought to underlie memory^53^, a function that is likely paralleled by VGF^54^. Hence, it is possible that the Cepo-induced increases in dorsal dentate BDNF/TH and ventral CA1 VGF may have promoted rescue of working memory deficits in social defeated rats seen here.

Cepo appeared to accelerate acquisition of fear extinction in defeated rats, which showed reduced freezing that was reflected by a combination of increased BDNF, TH, and Arc in the dorsal dentate. In contrast, a combination of VGF, Arc, and neuritin in the dorsal dentate and CA1 was negatively correlated with changes in fear extinction, with dentate neuritin and both VGF and neuritin in the CA1 being significant predictors. Fear extinction was also negatively correlated with VGF in the mPFC. Similarly enhanced fear extinction appeared to be exhibited by Cepo-treated controls, which again was associated with increases in dentate VGF and neuritin and mPFC VGF. However, the markedly reduced fear recall exhibited by control rats that received Cepo suggests that seemingly enhanced fear extinction was a result of failure to acquire a learned conditioned fear response. In contrast, we posit that the greater fear recall and subsequent enhanced fear extinction shown by Cepo-treated defeated rats reflects heightened learning and memory formation in this group.

The transcriptional mechanisms involved in Cepo-induced gene regulation are worthy of further investigation. Four doses of Cepo resulted in gene expression changes that were maintained for more than two weeks. It is likely that Cepo causes intracellular signal transduction changes that activate key transcription factors, which elevate gene transcription in a sustained manner. Epo clinical trials in treatment resistant depressed patients have shown that cognitive improvement is sustained at least six weeks after the last dose^16^. If stable changes in gene expression are involved in the behavioral effects, it is quite likely that Cepo will also be capable of producing lasting improvement in behavior. Our results indicate that Cepo may be recommended for improving long-term memory in individuals without traumatic experiences, whereas all patients would appear to benefit from a combination of Cepo and psychotherapy when rescuing working memory deficits or extinguishing specific negative thoughts and behaviors.

The importance of treating cognitive dysfunction in depression is being appreciated with increasing concern as these deficits continue to compromise quality of life even after remission^55^. In a recent clinical trial involving over 1000 patients, three widely prescribed antidepressants were tested for efficacy in five cognitive domains^9^. None of the antidepressants produced improvement in any of the tested measures. Given the promising results obtained with Epo^15,16,46^, albeit in much smaller trials, but in multiple disorders, it is worthwhile to consider further research into safer alternatives, such as Cepo that have no hematological consequences. Moreover, the fact that the mechanism of action is trophic and via specific high affinity receptors, it could be employed as an add-on without interfering with current monoamine based drugs.

## Electronic supplementary material

Supplementary Materials 1

Supplementary Materials 2

Supplementary figure

Supplementary Tables
