# Supplementary material for: Carbamoylated erythropoietin modulates cognitive outcomes of social defeat and differentially regulates gene expression in the dorsal and ventral hippocampus
Source: Transl Psychiatry. 2018 Jun 8;8:113. doi: 10.1038/s41398-018-0168-9 (PMC5993867; doi:10.1038/s41398-018-0168-9)
Supplement: Supplementary file 1 — Supplementary Materials 1 [file 41398_2018_168_MOESM1_ESM.pdf]

Supplementary Methods

Hematocrit measurement

Epo, Cepo and vehicle were administered to male and female BALB/c mice (Envigo) at a dose of 33 μg/kg/day for 10 days. Five hours after the last dose trunk blood was collected into K2EDTA tubes (Greiner Bio-One) and then drawn into capillary tubes, sealed (Leica Critoseal) and spun in a capillary centrifuge. Hematocrit levels were determined by superimposing the capillaries on a graded reference chart.

Sample preparation for mass spectrometry analysis

Purified Cepo was re-suspended in ammonium bicarbonate (Sigma-Aldrich, St. Louis, MO) (for trypsin) or urea (Sigma-Aldrich) and ammounium bicarbonate buffer (for chymotrypsin), and reduced with DTT (Promega, Madison, WI) at 95°C for 5 min. Sample was cooled to room temperature and alkylated using iodoacetemide (Sigma-Aldrich) with incubation of 20min in the dark. Trypsin and chymotrypsin (both Promega) were added to individual samples at a 1:25 enzyme:protein (wt/wt) ratio and digested at 37°C for overnight. Following digestion, samples were cleaned using C18 ZipTip (Millipore, Billerica, MA), dried in speed vacuum and re-suspended in 0.1% formic acid (Honeywell Burdick & Jackson).

Mass spectrometry analysis

Each digest was analyzed using two LC-MS/MS systems: Eksigent cHiPLC column (75μm x 15cm ChromXP C-18-CL 3μm 120 Å) interfaced with 5600 Triple TOF (SCIEX) or nano-liquid chromatography system (TEMPO ™nano MDLC System, AB SCIEX, Framingham, MA, USA) equipped with two alternating peptide traps and a

PicoFrit RP-C18 column (New Objectives, Woburn, MA, USA) interfaced with Orbitrap ELITE ETD (ThermoFisher). Both systems ran a 60min linear gradient of 0-60% acetonitrile in 0.1% formic acid. Following acquisition, data were analyzed using PEAKS Studio v. 6 (Waterloo, ON, Canada) with human UniProt-Swiss-Prot database with the following settings: tolerance of parent ion was set at 10ppm, tolerance of fragment ion was set at 0.8Da, maximum 2 missed cleavages, peptide confidence level 99% (0.01 FDR). Variable PTMs specified during search include: oxidation (15.99Da; M), carboamidomethyl (57.02Da; C) and carbamoylation (43Ka; K; X@N where X equals any amino acid).

Social Defeat Stress

Briefly, experimental subjects were placed in the home cage of a larger aggressive male rat and allowed to interact physically for 10 min. Following this, subject rats were isolated behind a wire mesh barrier for 25 min to prevent further physical contact but allow transmission of auditory, olfactory, and visual cues. Intruder rats were confronted with a different resident male during each trial to control for variance in defeat intensity. Age-matched male controls (n=20) were placed in a novel empty cage for the duration of each defeat trial to control for handling and novel environment stress.

Novel Object Recognition

Rats were first habituated to novel opaque plastic testing arenas (52×33.5×30 cm) for 30 min. Acquisition trials were conducted the following day, with subjects placed for 10 min into the testing arena, which contained two identical objects (either 4 cm diameter plastic golf balls or 8 x 1.5 cm Nylabones®) positioned at opposite ends of the arena. Twenty four hours later, subjects were allowed to interact with one familiar

object from the previous day and one novel object for 10 min, with object exploration defined as the subject's nose being within 2 cm of the object. Sets of familiar and novel objects and their location in the testing arena were randomized across treatment groups to avoid biases towards object type and spatial location. The time spent exploring each object was measured using Noldus Ethovision XT 8, and used to calculate a single discrimination index ([time with novel – time with familiar] / [time with novel + time with familiar]) for each subject.

Contextual fear conditioning

Rats were placed for two minutes in a foot shock chamber (30 x 30 cm, Noldus Information Technology) fitted with an overhead camera and housed in a sound-attenuating chamber (Med-Associates, St. Albans, VT, USA), and then received 10 electric shocks (0.75 mA, 2 s duration) delivered every 74 s through the testing chamber floor. Subjects remained in the chamber for two further minutes with no shocks delivered before being removed. On days 2, 3 and 4 of testing, rats were placed in the same chamber for 8 min, with no shocks delivered to determine the extent of contextual fear learning (recall test, day 2) and extinction of contextual fear conditioning (days 3-4). Video footage was later scored for freezing behavior using Noldus Observer XT 10 by an observer blind to treatment. Freezing behavior was defined as complete immobility except for minor movements required for respiration

RNA isolation, QC and QPCR

Briefly, RNA was isolated with a non-phenolic RNAqueous kit (Invitrogen, Thermo Fisher) and quantified using the Nanodrop spectrophotometer (Thermo). RNA quality was determined using the Nanoassay on the Bioanalyzer (Agilent). A master mix

containing SybrGreen (Invitrogen, Life Technologies) and gene specific primers (IDT) were employed for PCR amplification of reverse transcribed cDNA in a Realplex Mastercycler realtime PCR machine (Eppendorf). Product specificity was determined by melt curve analysis. The housekeeping genes, cyclophilin and GAPDH were used to normalize QPCR data.

Modeling Gene x Behavior Relationships – Potential relationships between changes in gene expression within each brain region and behavior were examined using separate multiple linear regressions. Genes selected for the regression analyses comprised those showing significant changes in expression within a given brain region, as identified by two way ANOVA. These were then set as independent variables against the behavior of interest (working memory, novel object recognition, conditioned fear recall, and conditioned fear extinction). Multiple regressions for each of these behaviors against gene expression within a given region were first carried out using the full combination of selected genes, with specific genes then removed systematically to identify the combination providing the best linear fit (Supplementary Table 1).

To gain insight into how gene expression across brain regions may explain behavioral variance, multiple linear regressions were again carried out (see Supplementary Material). First, combinations of the genes providing the best fit for each region against a particular behavior were identified from the within-region regression analyses, e.g., BDNF + TH in the dorsal dentate gyrus for working memory. The gene that was a significant predictor ($P<0.05$) within that combination was then chosen for inclusion in the across-region regressions. This was carried out for each gene x region combination that was shown from the within-region regressions to explain

variance in the given behavior, with products then incorporated into regression as independent variables (Supplementary Table 2), e.g., model for working memory = (dorsal dentate gyrus BDNF) + (dorsal CA1 VGF and TH) + (ventral CA1 Arc). For both within- and across region analyses, the number of possible combinations to be tested was calculated without replacement as n! / r! x (n-r)!, with n! being the factorial of the number of possible arrangements for a given set of candidates, e.g., VGF + TH + Arc + Nrn =4!, and r! the factorial of the number of redundancies for a set number of factors within these possible arrangements, e.g., just using 3 out of the 4 genes yields r! = 3!. All regressions were performed using SigmaPlot 13.0 at an alpha level of 0.05.

Supplementary Results

Within Brain Region Gene x Behavior Relationships

Dorsal DG - The combination of BDNF and TH in the dorsal DG provided the model of best fit to explain variance in working memory ($R2 = 0.32$, $P = 0.006$), with BDNF being the significant predictor in this model ($P = 0.006$). Fear learning behavior also showed significant relationships with gene expression in the dorsal DG. Variance in the amount of conditioned freezing during the Recall session was best explained by a combination of TH, Arc and neuritin ($R2 = 0.25$, $P = 0.048$), with TH being positively correlated with freezing ($P = 0.006$). As for working memory, the combination of genes provided the strongest model for freezing during Recall, with TH alone producing a non-significant correlation ($R2 = 0.112$, $P = 0.07$). Similarly, the model produced by the combination of BDNF, TH and Arc ($R2 = 0.31$, $P = 0.02$) for explaining the degree of freezing in the first Extinction session, in which only BDNF was a significant negative predictor ($P = 0.034$),

was stronger than when BDNF alone was regressed against behavior ($R2 = 0.22$, $P = 0.02$). Interestingly, the model produced by the combination of VGF, Arc and neuritin also accounted for variance in fear extinction ($R2 = 0.35$, $P = 0.01$), with neuritin negatively correlated with freezing ($P = 0.03$). Again, this was stronger than when neuritin alone was regressed against behavior ($R2 = 0.26$, $P = 0.02$). None of the 31 possible combinations of genes in the dorsal DG were significantly correlated with performance in the novel object recognition task.

Ventral DG – The only behavior showing any relationship with gene expression in this region was novel object recognition, with neuritin alone being negatively correlated with performance in this task (Fig. 5b, $R2 = 0.26$, $P = 0.007$). The strength of this model was not increased with inclusion of additional genes.

Dorsal CA1 – No combination of genes in the dorsal CA1 accounted for variance in working memory. However, VGF alone was found to be positively correlated with working memory performance ($R2 = 0.19$, $P = 0.02$). Independent of this, TH alone showed a relationship of similar strength with working memory ability ($R2 = 0.18$, $P = 0.02$), but was negatively correlated. The only other behavior associated with gene expression in this region was fear extinction, where the combination of VGF, Arc and neuritin accounted for variance in freezing ($R2 = 0.34$, $P = 0.013$). In this model, both VGF ($P = 0.009$) and neuritin ($P = 0.047$) were negatively correlated with expression of freezing behavior.

Ventral CA1 – There was a weak relationship between working memory and Arc alone in the ventral CA1 ($R2 = 0.13$, $P = 0.045$), with no gene combination being significant. Expression of VGF and Arc combined provided the strongest model to explain the

degree of freezing during the first fear extinction session ($R^2 = 0.32$, $P = 0.007$), in which Arc was positively correlated with freezing behavior ($P = 0.004$). No gene combination in the ventral CA1 was significantly associated with either novel object recognition performance or conditioned fear behavior during the Recall session.

mPFC – The only behavior associated with gene expression in the mPFC was fear extinction, with a combination of BDNF, VGF and TH providing the model of best fit ($R^2 = 0.27$, $P = 0.043$), in which VGF showed a negative correlation with freezing expression ($P = 0.013$).
