# Supplementary material for: Carbamoylated erythropoietin modulates cognitive outcomes of social defeat and differentially regulates gene expression in the dorsal and ventral hippocampus
Source: Transl Psychiatry. 2018 Jun 8;8:113. doi: 10.1038/s41398-018-0168-9 (PMC5993867; doi:10.1038/s41398-018-0168-9)
Supplement: Supplementary file 2 — Supplementary Materials 2 [file 41398_2018_168_MOESM2_ESM.pdf]

CEPO-Chymotrypsin Orbitrap

	Peptide	Mass	m/z	UniProt ID
1	L.RALGAQK(+43.00)EAISPPDAASAAPL.R	2076.0906	1039.0551	P01588|EPO_HUMAN
2	W.KRMEVGQQAVEVW.Q	1558.7926	780.4032	P01588|EPO_HUMAN
3	W.K(+43.00)RMEVGQQAVEVW.Q	1601.7926	801.9067	P01588|EPO_HUMAN
4	Y.LLEAK(+43.00)EAENITTGC(+57.02)AEH.C	1927.8887	964.9548	P01588|EPO_HUMAN
5	L.EAK(+43.00)EAENITTGC(+57.02)AEH.C	1701.7206	851.8702	P01588|EPO_HUMAN
6	L.QLHVDKAVSGL.R	1165.6455	583.8301	P01588|EPO_HUMAN
7	L.RTITADTF.R	923.4713	462.7431	P01588|EPO_HUMAN
8	Y.AWK(+43.00)RM(+15.99)EVGQQAVEVW.Q	1874.9039	938.4609	P01588|EPO_HUMAN
9	K.RMEVGQQAVEVW.Q	1430.6976	716.3568	P01588|EPO_HUMAN
10	W.K(+43.00)RM(+15.99)EVGQQAVEVW.Q	1617.7875	809.9025	P01588|EPO_HUMAN
11	W.KRM(+15.99)EVGQQAVEVW.Q	1574.7875	788.4003	P01588|EPO_HUMAN
12	L.R(+43.00)ALGAQKEAISPPDAASAAPL.R	2076.0906	1039.0547	P01588|EPO_HUMAN
13	L.QLHVDK(+43.00)AVSGLRSL.T	1564.8627	783.4409	P01588|EPO_HUMAN
14	L.E(+43.00)AKEAENITTGC(+57.02)AEH.C	1701.7206	851.8704	P01588|EPO_HUMAN
15	W.K(+43.00)(+43.00)RMEVGQQAVEVW.Q	1644.7926	823.409	P01588|EPO_HUMAN
16	L.LEAK(+43.00)EAENITTGC(+57.02)AEH.C	1814.8047	908.4128	P01588|EPO_HUMAN
17	P.LRTITADTF.R	1036.5553	519.285	P01588|EPO_HUMAN
18	Y.A(+43.00)WKRM(+15.99)EVGQQAVEVW.Q	1874.9039	938.4626	P01588|EPO_HUMAN
19	L.RALGAQK(+43.00)EAISPPD.A	1494.7732	748.3962	P01588|EPO_HUMAN
20	P.PRLIC(+57.02)DSRVL.E	1227.6758	410.2328	P01588|EPO_HUMAN
21	L.RALGAQK(+43.00)EAISPPDAA.S	1636.8474	819.434	P01588|EPO_HUMAN
22	G.APPRLIC(+57.02)DSRVL.E	1395.7656	466.2626	P01588|EPO_HUMAN
23	Y.AWK(+43.00)RMEVGQQAVEVW.Q	1858.9089	930.4649	P01588|EPO_HUMAN
24	L.QLHVDK(+43.00)AVSGLR.S	1364.7466	683.3835	P01588|EPO_HUMAN
25	R.LIC(+57.02)DSRVLERY.L	1422.7289	712.3715	P01588|EPO_HUMAN
26	L.ALLSEAVL.R	814.48	408.2472	P01588|EPO_HUMAN
27	L.RALGAQK(+43.00)EAISPPDAAS.A	1723.8794	862.9494	P01588|EPO_HUMAN
28	L.IC(+57.02)DSRVL.E	861.4378	431.7262	P01588|EPO_HUMAN
29	Y.SNFLRGK(+43.00)L.K	976.5396	489.28	P01588|EPO_HUMAN
30	L.QLHVDK(+43.00)AVSGL.R	1208.6455	605.3318	P01588|EPO_HUMAN
31	L.GAQK(+43.00)EAISPPDAASAAPL.R	1735.8682	868.9446	P01588|EPO_HUMAN
32	A.PPRLIC(+57.02)DSRVL.E	1324.7285	442.5838	P01588|EPO_HUMAN
33	R.MEVGQQAVEVW.Q	1274.5966	638.3054	P01588|EPO_HUMAN
34	L.GAPPRLIC(+57.02)DSRVL.E	1452.7871	485.2699	P01588|EPO_HUMAN
35	W.K(+43.00)(+43.00)RM(+15.99)EVGQQAVEVW.Q	1660.7875	831.4065	P01588|EPO_HUMAN
36	L.RALGAQK(+43.00)EAISPPDA.A	1565.8103	783.9153	P01588|EPO_HUMAN
37	M.EVGQQAVEVW.Q	1143.556	572.7855	P01588|EPO_HUMAN
38	W.K(+43.00)RM(+15.99)EVGQQAVEVWQGL.A	1915.9515	958.9853	P01588|EPO_HUMAN
39	P.RLIC(+57.02)DSRVL.E	1130.623	566.3187	P01588|EPO_HUMAN
40	L.R(+43.00)ALGAQKEAISPPDA.A	1565.8103	783.9127	P01588|EPO_HUMAN
41	L.RSLTTLL.R	802.4912	402.253	P01588|EPO_HUMAN
42	R.LIC(+57.02)DSRVL.E	974.5219	488.2688	P01588|EPO_HUMAN
43	W.K(+43.00)RMEVGQQAVE.V	1316.6449	659.3324	P01588|EPO_HUMAN
44	L.G(+43.00)AQKEAISPPDAASAAPL.R	1735.8682	868.9448	P01588|EPO_HUMAN
45	K.RM(+15.99)EVGQQAVEVW.Q	1446.6925	724.353	P01588|EPO_HUMAN

46	W.K(+43.00)RMEVGQQAVEV.W	1415.7133	708.8671	P01588|EPO_HUMAN
47	W.EPLQLHVDK(+43.00)AVSGL.R	1547.825	774.9223	P01588|EPO_HUMAN
48	L.K(+43.00)LYTGEAC(+57.02)RTGD.R	1412.6296	707.3245	P01588|EPO_HUMAN
49	L.HVDK(+43.00)AVSGL.R	967.5029	484.7619	P01588|EPO_HUMAN
50	L.IC(+57.02)DSRVLERY.L	1309.6449	437.5559	P01588|EPO_HUMAN
51	L.H(+43.00)VDKAVSGL.R	967.5029	484.7615	P01588|EPO_HUMAN
52	L.G(+43.00)APPRLICDSRVLERY.L	1886.9727	629.9998	P01588|EPO_HUMAN
53	L.R(+43.00)TITADTF.R	966.4713	484.2454	P01588|EPO_HUMAN
54	L.R(+43.00)ALGAQK(+43.00)EAISPPDAASAAPL.R	2119.0906	1060.5585	P01588|EPO_HUMAN
55	L.RSLTTL.L	689.4072	345.7104	P01588|EPO_HUMAN
56	G.A(+43.00)PPRLIC(+57.02)DSRVL.E	1438.7656	720.3933	P01588|EPO_HUMAN
57	L.RALGAQKEAISPPDAASAAPL.R	2033.0905	678.7039	P01588|EPO_HUMAN
58	G.A(+43.00)PPRLIC(+57.02)DSRVLERY.L	1886.9727	630.0002	P01588|EPO_HUMAN
59	P.V(+43.00)LGAPPRLIC(+57.02)DSRVL.E	1707.9396	854.9793	P01588|EPO_HUMAN

CEPO-Chymotrypsin-TTOF

	Peptide	Mass	m/z	UniProt ID
1	L.QLHVDKAVSGL.R	1165.6455	583.8337	P01588|EPO_HUMAN
2	Y.LLEAK(+43.00)EAENITTGC(+57.02)AEHC(+57.02).S	2087.9194	696.9843	P01588|EPO_HUMAN
3	L.RALGAQK(+43.00)EAISPPDAA.S	1636.8474	546.6265	P01588|EPO_HUMAN
4	L.RTITADTF.R	923.4713	462.7452	P01588|EPO_HUMAN
5	W.K(+43.00)RMEVGQQAVEVW.Q	1601.7926	801.9067	P01588|EPO_HUMAN
6	L.SEAVLRGQAL.L	1042.577	522.2976	P01588|EPO_HUMAN
7	L.E(+43.00)AKEAENITTGC(+57.02)AEH.C	1701.7206	568.2488	P01588|EPO_HUMAN
8	L.HVDKAVSGL.R	924.5029	463.2623	P01588|EPO_HUMAN
9	M.EVGQQAVEVW.Q	1143.556	572.7894	P01588|EPO_HUMAN
10	L.RALGAQKEAISPPDAASAAPL.R	2033.0905	678.7081	P01588|EPO_HUMAN
11	G.APPRLIC(+57.02)DSRVL.E	1395.7656	466.2668	P01588|EPO_HUMAN
12	Y.SNFLRGK(+43.00)L.K	976.5396	489.2813	P01588|EPO_HUMAN
13	P.PRLIC(+57.02)DSRVL.E	1227.6758	410.234	P01588|EPO_HUMAN
14	K.RMEVGQQAVEVW.Q	1430.6976	716.3624	P01588|EPO_HUMAN
15	Y.LLEAK(+43.00)EAENITTGC(+57.02)AEH.C	1927.8887	964.9509	P01588|EPO_HUMAN
16	Y.AWK(+43.00)RM(+15.99)EVGQQAVEVW.Q	1874.9039	938.4638	P01588|EPO_HUMAN
17	A.PPRLIC(+57.02)DSRVL.E	1324.7285	663.3764	P01588|EPO_HUMAN
18	L.RALGAQK(+43.00)EAISPPDAASAAPL.R	2076.0906	693.0406	P01588|EPO_HUMAN
19	P.LRTITADTF.R	1036.5553	519.2876	P01588|EPO_HUMAN
20	L.LRALGAQK(+43.00)EAISPPDAASAAPL.R	2189.1746	730.7338	P01588|EPO_HUMAN
21	W.K(+43.00)RM(+15.99)EVGQQAVEVW.Q	1617.7875	809.9075	P01588|EPO_HUMAN
22	W.KRMEVGQQAVEVW.Q	1558.7926	780.4088	P01588|EPO_HUMAN
23	R.MEVGQQAVEVW.Q	1274.5966	638.3113	P01588|EPO_HUMAN
24	L.GAPPRLIC(+57.02)DSRVL.E	1452.7871	485.2715	P01588|EPO_HUMAN
25	L.Y(+43.00)TGEAC(+57.02)RTGD.R	1171.4506	586.7379	P01588|EPO_HUMAN
26	W.K(+43.00)RMEVGQQAVE.V	1316.6449	659.3342	P01588|EPO_HUMAN
27	L.QLHVDK(+43.00)AVSGL.R	1208.6455	605.3351	P01588|EPO_HUMAN
28	R.LIC(+57.02)DSRVL.E	974.5219	488.2706	P01588|EPO_HUMAN
29	G.A(+43.00)PPRLIC(+57.02)DSRVL.E	1438.7656	720.3954	P01588|EPO_HUMAN
30	L.RALGAQK(+43.00)EAI.S	1098.6086	550.3147	P01588|EPO_HUMAN
31	L.H(+43.00)VDKAVSGLRSL.T	1323.7201	442.2516	P01588|EPO_HUMAN
32	L.K(+43.00)LYTGEAC(+57.02)RTGD.R	1412.6296	707.328	P01588|EPO_HUMAN
33	W.KRM(+15.99)EVGQQAVEVW.Q	1574.7875	525.9403	P01588|EPO_HUMAN
34	L.HVDK(+43.00)AVSGL.R	967.5029	484.7619	P01588|EPO_HUMAN
35	Y.LLEAK(+43.00)EAEN.I	1058.5186	530.2699	P01588|EPO_HUMAN
36	L.RALGAQK(+43.00)EAISPPD.A	1494.7732	748.4007	P01588|EPO_HUMAN
37	L.IC(+57.02)DSRVLERY.L	1309.6449	437.5543	P01588|EPO_HUMAN
38	K.RM(+15.99)EVGQQAVEVW.Q	1446.6925	724.3592	P01588|EPO_HUMAN
39	L.Q(+43.00)LHVDKAVSGL.R	1208.6455	605.3358	P01588|EPO_HUMAN
40	W.K(+43.00)RMEVGQQAVEV.W	1415.7133	708.8704	P01588|EPO_HUMAN
41	E.AISPPDAASAAPL.R	1179.6135	590.8161	P01588|EPO_HUMAN
42	K.R(+43.00)M(+15.99)EVGQQAVEVW.Q	1489.6925	745.8593	P01588|EPO_HUMAN
43	L.RSLTTLL.R	802.4912	402.2565	P01588|EPO_HUMAN
44	P.RLIC(+57.02)DSRVL.E	1130.623	566.3233	P01588|EPO_HUMAN
45	Y.AWK(+43.00)RMEVGQQAVEVW.Q	1858.9089	930.4692	P01588|EPO_HUMAN

46	L.IC(+57.02)DSRVL.E	861.4378	431.7258	P01588|EPO_HUMAN
47	L.ALLSEAVL.R	814.48	815.4922	P01588|EPO_HUMAN
48	L.R(+43.00)TITADTF.R	966.4713	484.2461	P01588|EPO_HUMAN
49	K.R(+43.00)MEVGQQAVEVW.Q	1473.6976	737.8594	P01588|EPO_HUMAN
50	Q.LHVDK(+43.00)AVSGL.R	1080.5869	541.3053	P01588|EPO_HUMAN
51	L.GAQK(+43.00)EAISPPDAASAAPL.R	1735.8682	868.9471	P01588|EPO_HUMAN
52	C.DSRVLERY.L	1036.5302	519.273	P01588|EPO_HUMAN
53	L.RALGAQK(+43.00)EAIS.P	1185.6407	593.8331	P01588|EPO_HUMAN
54	G.A(+43.00)PPRLICDSRVL.E	1381.7441	691.8847	P01588|EPO_HUMAN
55	G.L(+43.00)PVLGAPPRLIC(+57.02)DSRVL.E	1918.0764	640.3705	P01588|EPO_HUMAN
56	R.LIC(+57.02)DSRVLERY.L	1422.7289	475.253	P01588|EPO_HUMAN
57	L.GAQK(+43.00)EAISPPD.A	1154.5509	578.2883	P01588|EPO_HUMAN
58	H.V(+43.00)DKAVSGL.R	830.444	416.2319	P01588|EPO_HUMAN
59	Y.A(+43.00)WKRM(+15.99)EVGQQAVEVW.Q	1874.9039	938.4626	P01588|EPO_HUMAN
60	L.GAQK(+43.00)EAISPPDAA.S	1296.6251	649.3192	P01588|EPO_HUMAN
61	W.EPLQLHVDK(+43.00)AVSGL.R	1547.825	774.9254	P01588|EPO_HUMAN

CEPO-Trypsin-Orbitrap

	Peptide	Mass	m/z	UniProt ID
1	R.ALGAQK(+43.00)EAISPPDAASAAPLR.T	2076.0906	1039.0552	P01588|EPO_HUMAN
2	K.EAISPPDAASAAPLR.T	1464.7572	733.3871	P01588|EPO_HUMAN
3	R.M(+15.99)EVGQQAVEVWQGLALLSEAVLR.G	2541.3262	1271.67	P01588|EPO_HUMAN
4	R.A(+43.00)LGAQKEAISPPDAASAAPLR.T	2076.0906	1039.0559	P01588|EPO_HUMAN
5	R.MEVGQQAVEVWQGLALLSEAVLR.G	2525.3311	1263.673	P01588|EPO_HUMAN
6	R.G(+43.00)KLKLYTGEAC(+57.02)R.T	1437.734	719.8752	P01588|EPO_HUMAN
7	R.G(+43.00)KLK(+43.00)LYTGEAC(+57.02)R.T	1480.734	741.3771	P01588|EPO_HUMAN
8	A.ISPPDAASAAPLR.T	1264.6775	633.3461	P01588|EPO_HUMAN
9	R.GK(+43.00)LK(+43.00)LYTGEAC(+57.02)R.T	1480.734	741.3795	P01588|EPO_HUMAN
10	W.QGLALLSEAVLR.G	1268.7452	635.3799	P01588|EPO_HUMAN
11	R.VYSNFLR.G	897.4708	449.7433	P01588|EPO_HUMAN
12	K.LK(+43.00)LYTGEAC(+57.02)R.T	1252.6176	627.3191	P01588|EPO_HUMAN
13	R.GK(+43.00)LK(+43.00)LYTGEACR.T	1423.7125	712.8674	P01588|EPO_HUMAN
14	K.VNFYAWK.R	926.465	464.2397	P01588|EPO_HUMAN
15	K.VNFYAWK(+43.00)R.M	1125.5662	563.793	P01588|EPO_HUMAN
16	Q.GLALLSEAVLR.G	1140.6866	571.3506	P01588|EPO_HUMAN
17	K.RM(+15.99)EVGQQAVEVWQGLALLSEAVLR.G	2697.4272	900.1496	P01588|EPO_HUMAN
18	R.TITADTFR.K	923.4713	462.7432	P01588|EPO_HUMAN
19	R.G(+43.00)KLK(+43.00)LYTGEACR.T	1423.7125	712.8703	P01588|EPO_HUMAN
20	R.LIC(+57.02)DSRVLER.Y	1259.6655	630.8408	P01588|EPO_HUMAN
21	Q.QAVEVWQGLALLSEAVLR.G	1981.0996	661.3741	P01588|EPO_HUMAN
22	Q.K(+43.00)EAISPPDAASAAPLR.T	1635.8522	818.937	P01588|EPO_HUMAN
23	Q.AVEVWQGLALLSEAVLR.G	1853.041	618.6879	P01588|EPO_HUMAN
24	R.G(+43.00)K(+43.00)LK(+43.00)LYTGEAC(+57.02)R.T	1523.734	762.8809	P01588|EPO_HUMAN
25	Q.PWEPLQLHVDK(+43.00)AVSGLR.S	1987.0581	663.3618	P01588|EPO_HUMAN
26	R.GK(+43.00)LKLYTGEAC(+57.02)R.T	1437.734	480.2539	P01588|EPO_HUMAN
27	L.ALLSEAVLR.G	970.5811	486.298	P01588|EPO_HUMAN
28	R.MEVGQQAVEVWQGLAL.L	1756.8818	879.4483	P01588|EPO_HUMAN
29	E.AISPPDAASAAPLR.T	1335.7146	668.8643	P01588|EPO_HUMAN
30	W.Q(+43.00)GLALLSEAVLR.G	1311.7452	656.8825	P01588|EPO_HUMAN
31	R.GKLK(+43.00)LYTGEAC(+57.02)R.T	1437.734	480.2542	P01588|EPO_HUMAN
32	R.M(+15.99)EVGQQAVEVWQGLAL.L	1772.8767	887.4471	P01588|EPO_HUMAN
33	R.A(+43.00)LGAQK(+43.00)EAISPPDAASAAPLR.T	2119.0906	1060.557	P01588|EPO_HUMAN
34	G.LALLSEAVLR.G	1083.6652	542.8396	P01588|EPO_HUMAN
35	R.MEVGQQAVEVWQGL.A	1572.7606	787.3871	P01588|EPO_HUMAN
36	K.L(+43.00)KLYTGEAC(+57.02)R.T	1252.6176	627.3195	P01588|EPO_HUMAN
37	R.G(+43.00)K(+43.00)LKLYTGEAC(+57.02)R.T	1480.734	741.3798	P01588|EPO_HUMAN
38	R.LIC(+57.02)DSR.V	762.3694	382.1919	P01588|EPO_HUMAN
39	N.SSQPWEPLQLHVDK(+43.00)AVSGLR.S	2289.1807	1145.5997	P01588|EPO_HUMAN
40	G.K(+43.00)LK(+43.00)LYTGEAC(+57.02)R.T	1423.7125	712.8691	P01588|EPO_HUMAN
41	R.SLTTLLR.A	802.4912	402.2531	P01588|EPO_HUMAN
42	V.YSNFLR.G	798.4024	400.2087	P01588|EPO_HUMAN
43	G.L(+43.00)PVLGAPPR.L	961.5651	481.7926	P01588|EPO_HUMAN
44	R.M(+15.99)(+43.00)EVGQQAVEVWQGLALLSEAVLR.G	2584.3262	862.4505	P01588|EPO_HUMAN
45	G.QQAVEVWQGLALLSEAVLR.G	2109.1582	704.06	P01588|EPO_HUMAN
46	L.HVDK(+43.00)AVSGLR.S	1123.604	562.8121	P01588|EPO_HUMAN
47	R.S(+43.00)LTTLLR.A	845.4912	423.7556	P01588|EPO_HUMAN

48	R.Y(+43.00)LLEAKEAENITTGC(+57.02)AEH.C	2090.9521	1046.4832	P01588|EPO_HUMAN
49	L.A(+43.00)LLSEAVLR.G	1013.5811	507.8001	P01588|EPO_HUMAN
50	R.A(+43.00)LGAQKEAISPPD.A	1338.6721	670.3461	P01588|EPO_HUMAN
51	R.ALGAQK(+43.00)EAISPPD.A	1338.6721	670.3459	P01588|EPO_HUMAN
52	S.LTTLLR.A	715.4592	358.7368	P01588|EPO_HUMAN
53	Y.TGEAC(+57.02)R.T	692.2911	347.153	P01588|EPO_HUMAN
54	R.M(+15.99)EVGQQAVEVWQGL.A	1588.7555	795.385	P01588|EPO_HUMAN
55	W.EPLQLHVDK(+43.00)AVSGLR.S	1703.926	568.9848	P01588|EPO_HUMAN
56	R.YLLEAK(+43.00)EAENITTGC(+57.02)AEH.C	2090.9521	1046.4858	P01588|EPO_HUMAN
57	R.V(+43.00)YSNFLR.G	940.4708	471.2461	P01588|EPO_HUMAN
58	Q.L(+43.00)HVDKAVSGLR.S	1236.688	413.2386	P01588|EPO_HUMAN
59	N.FYAWK(+43.00)R.M	912.4548	457.2361	P01588|EPO_HUMAN
60	L.K(+43.00)LYTGEAC(+57.02)R.T	1139.5334	570.7762	P01588|EPO_HUMAN
