# Supplementary figures and images for: Carbamoylated erythropoietin modulates cognitive outcomes of social defeat and differentially regulates gene expression in the dorsal and ventral hippocampus
Source: Transl Psychiatry. 2018 Jun 8;8:113. doi: 10.1038/s41398-018-0168-9 (PMC5993867; doi:10.1038/s41398-018-0168-9)

Supplementary Figure 1

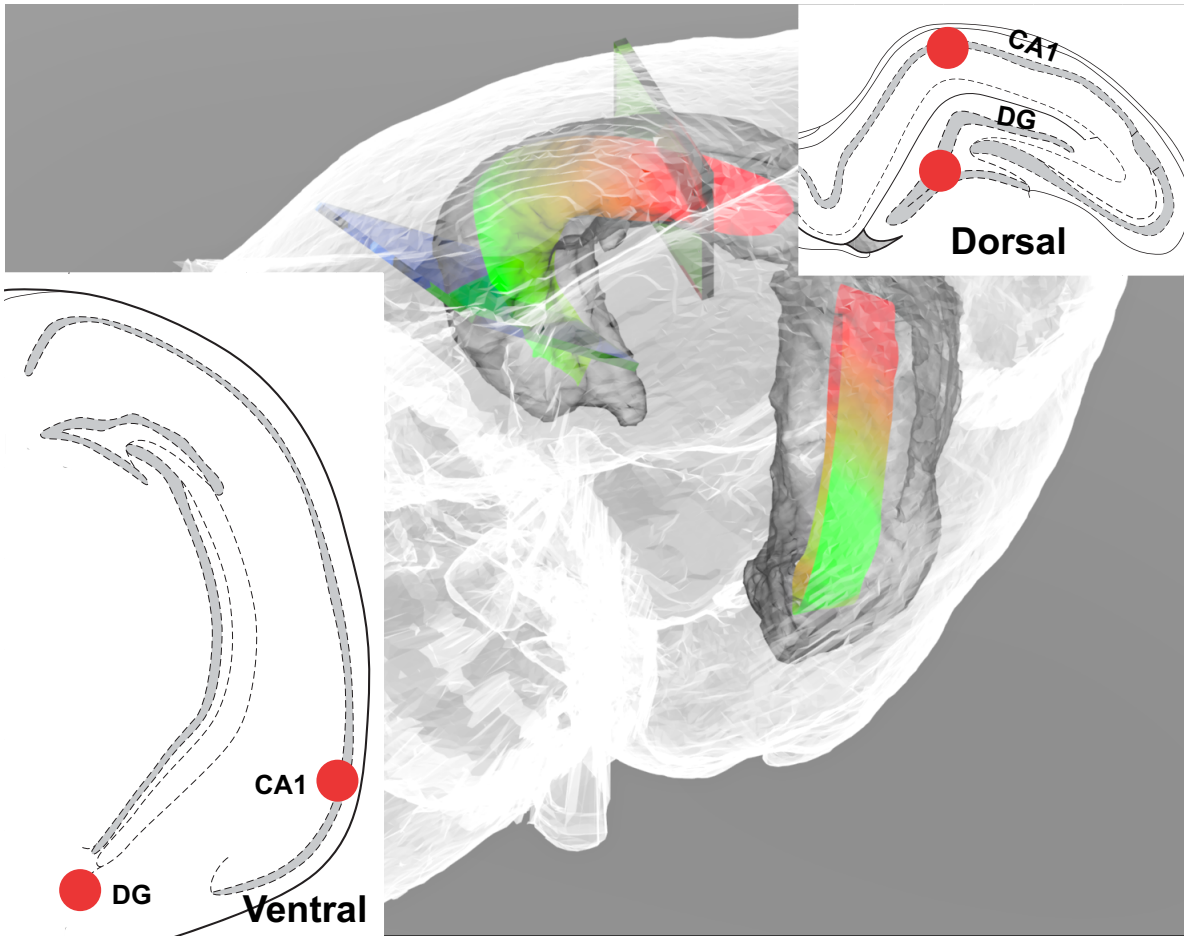

Supplement: Supplementary file 3 — Supplementary figure [file 41398_2018_168_MOESM3_ESM.pdf]
