# Supplementary material for: Carbamoylated erythropoietin modulates cognitive outcomes of social defeat and differentially regulates gene expression in the dorsal and ventral hippocampus
Source: Transl Psychiatry. 2018 Jun 8;8:113. doi: 10.1038/s41398-018-0168-9 (PMC5993867; doi:10.1038/s41398-018-0168-9)
Supplement: Supplementary file 4 — Supplementary Tables [file 41398_2018_168_MOESM4_ESM.pdf]

Table 1. Genes within specific brain regions chosen for multiple linear regressions against behavior.

Brain region	Candidate genes	Number of possible combinations with varying numbers of genes (one or more genes)
Dorsal dentate gyrus	BDNF, VGF, TH, Arc, Nrn	31
Ventral dentate gyrus	Arc, Nrn	4
Dorsal CA1	VGF, TH, Arc, Nrn	15
Ventral CA1	VGF, TH, Arc	7
Medial prefrontal cortex	BDNF, VGF, TH, Arc	15

Table 2. Gene expression changes across brain regions chosen for multiple linear regressions against specific behaviors.

Behavior	Candidate region / gene combinations	Number of possible combinations (two or more candidates)
Working memory	Dorsal dentate gyrus BDNF<br>Dorsal CA1 VGF + TH<br>Ventral CA1 Arc	11
Novel Object Recognition	Ventral dentate gyrus Arc + Nrn	1
Fear Recall	Dorsal dentate gyrus TH , Arc, Nrn	7
Fear Extinction	Dorsal dentate gyrus BDNF<br>Dorsal dentate gyrus Nrn<br>Dorsal CA1 VGF + Nrn<br>Ventral CA1 Arc<br>Medial prefrontal cortex VGF	57

Table 3. Gene combinations providing models of best fit for multiple linear regressions of gene expression within each brain region against specific behaviors. The gene(s) identified as significant predictors in each model are also listed.

Behavior	Working memory (Discrimination index)	Novel Object Recognition (Discrimination index)	Conditioned fear (Recall session - time freezing)	Fear Extinction (first session - time freezing)
Dorsal DG	**BDNF & TH**<br>$R^2 = 0.32$ (+)<br>$F(2,28) = 6.19$<br>$p = 0.006$<br>*Predictor(s)*: BDNF<br>($p = 0.006$)	None	**TH, Arc & Nrn**<br>$R^2 = 0.25$ (+)<br>$F(3,27) = 3.01$<br>$p = 0.048$<br>*Predictor(s)*: TH<br>($p = 0.006$)	*Model 1*: **BDNF, TH & Arc**<br>$R^2 = 0.31$ (+)<br>$F(3,26) = 3.84$<br>$p = 0.021$<br>Predictor(s): BDNF<br>($p = 0.034$)<br><br>*Model 2*: **VGF, Arc & Nrn**<br>$R^2 = 0.35$ (-)<br>$F(3,26) = 4.67$<br>$p = 0.01$<br>*Predictor(s)*: Nrn<br>($p = 0.03$)
Ventral DG	None	None	**Nrn alone**<br>$R^2 = 0.26$ (-)<br>$F(1,25) = 8.52$<br>$p = 0.007$	None
Dorsal CA1	*Model 1*: **VGF alone**<br>$R^2 = 0.13$ (+)<br>$F(1,29) = 6.26$<br>$p = 0.018$<br><br>*Model 2*: **TH alone**<br>$R^2 = 0.18$ (-)<br>$F(1,29) = 6.12$<br>$p = 0.02$	None	None	**VGF, Arc & Nrn**<br>$R^2 = 0.34$ (-)<br>$F(3,26) = 4.38$<br>$p = 0.015$<br>*Predictor(s)*: VGF<br>($p = 0.009$)<br>Nrn<br>($p = 0.047$)
Ventral CA1	**Arc alone**<br>$R^2 = 0.13$ (-)<br>$F(1,29) = 4.38$<br>$p = 0.045$	None	None	**VGF & Arc**<br>$R^2 = 0.31$ (+)<br>$F(2,27) = 6.1$<br>$p = 0.007$
mPFC	None	None	None	**BDNF, VGF & TH**<br>$R^2 = 0.27$ (-)<br>$F(3,26) = 3.07$<br>$p = 0.043$<br>*Predictor(s)*: VGF<br>($p = 0.013$)

(+) and (-) next to R2 values indicate positive and negative correlations respectively. BDNF = brain derived neurotrophic factor, VGF is non-acronymic, TH = tyrosine hydroxylase, Nrn = neuritin.
